# Committees of Legislation in Medical Societies

**Published:** 1898-09

**Authors:** 


### Committees of Legislation in Medical Societies.—Dr. Love's Views.

---

At a recent meeting of the Arkansas State Medical Society at Eureka Springs, Ark., which it was my pleasure to attend for one day, the opinion which I have long held, in opposition to medical societies making any efforts to control legislation by the appointment of committees for that purpose, was strengthened and confirmed.

The question was discussed quite a while, and I am in full sympathy with the sentiments of Dr. Welch of Fort Smith, Ark., an ex-president of the society, who said that "by our efforts in the majority of the States we have simply placed ourselves in an attitude of suppliants seeking special privileges and class legislation." [And been snubbed for our pains.—Daniel.]

The great trouble is that the great majority of the physicians are too busy even to attend to their own private business, in their devotion to their patients. They neglect their own interests the world over, and when it comes to doing work necessary along the lines of legislation in the States, they will not do it, and the legislation, which is secured, almost uniformly legalizes and protects quacks and mountebanks at the expense of the scientific part of the profession.

The truth of the matter is, that the well qualified physician needs no protection, and certainly the people have given evidence that they want none. "You cannot make a milk punch out of a sow's ear," nor can you pass any law, which will prevent the blessed people from patronizing frauds and fakirs. As long as they love to be hoodwinked and humbugged, there is no law that can prevent it.

Take for instance, the State of Missouri, and today, the properly educated graduated physician has to come under the control of the law, when the Osteopath, and the fraud in general, is above the law. So it will continue to be.

The properly educated members of the profession should devote themselves to the transaction of their own business, the care of their patients, and to the advancement of science, fully confident that in the final windup their places will be definitely fixed, and they will not need to dabble and hobnob with the skunks and scoundrels that go to make up the large part of many State Legislatures.—*Medical Mirror*, June, '98.

